# Beyond mechanical loading: The metabolic contribution of obesity in osteoarthritis unveils novel therapeutic targets

**DOI:** 10.1016/j.heliyon.2023.e15700

**Published:** 2023-04-25

**Authors:** Basma H. Sobieh, Hala O. El-Mesallamy, Dina H. Kassem

**Affiliations:** a Biochemistry Department, Faculty of Pharmacy, Ain Shams University, Cairo, Egypt; b Faculty of Pharmacy, Sinai University, Sinai, Egypt

**Keywords:** Osteoarthritis, Obesity, Dyslipidemia, Insulin resistance, Adipokines, Metrnl

## Abstract

Osteoarthritis (OA) is a prevalent progressive disease that frequently coexists with obesity. For several decades, OA was thought to be the result of ageing and mechanical stress on cartilage. Researchers’ perspective has been greatly transformed when cumulative findings emphasized the role of adipose tissue in the diseases. Nowadays, the metabolic effect of obesity on cartilage tissue has become an integral part of obesity research; hoping to discover a disease-modifying drug for OA. Recently, several adipokines have been reported to be associated with OA. Particularly, metrnl (meteorin-like) and retinol-binding protein 4 (RBP4) have been recognized as emerging adipokines that can mediate OA pathogenesis. Accordingly, in this review, we will summarize the latest findings concerned with the metabolic contribution of obesity in OA pathogenesis, with particular emphasis on dyslipidemia, insulin resistance and adipokines. Additionally, we will discuss the most recent adipokines that have been reported to play a role in this context. Careful consideration of these molecular mechanisms interrelated with obesity and OA will undoubtedly unveil new avenues for OA treatment.

## Highlights

• OA is a metabolic disease not just a tear and wear form of arthritis.
• Dyslipidemia, IR, and adipokines are multiple key players mediating obesity-OA interplay.
• Metrnl and RBP4 are novel adipokines that can mediate OA pathogenesis.
• MetaBolic contribution of obesity in OA unveils new avenues for OA treatment.

## Graphical abstract

**Figure:**
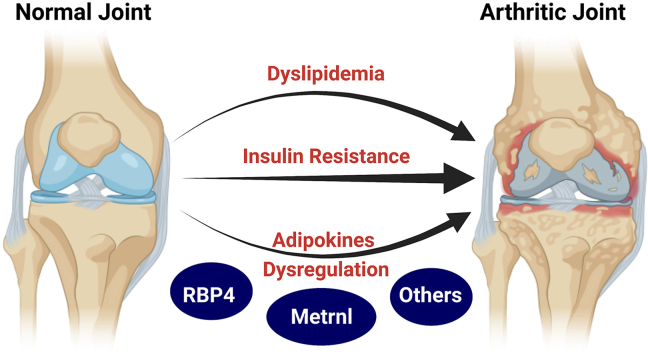

## 1 Introduction

Osteoarthritis (OA) is one of the most common joint disorders currently affecting about 528 million people globally [1]. It typically affects knees, hips, hands, spine, and feet, with a profound impact on several health outcomes [2]. Actually, arthritis has a tremendous influence on individuals; the physical impairment resulting from pain and loss of functional capability reduces the quality of life and increases the incidence of further morbidity. Although there are several gadgets and palliative medications available that can alleviate pain and enhance the quality of life, there is no pharmaceutical product that can hinder or reverse the onset of OA [3]. These palliative medicines have a great cost over the long term besides their side effects such as nephrotoxicity and gastric complications [4]. Nowadays, scientists are focusing on using hyaluronic acid (HA), platelet-rich plasma (PRP), mesenchymal stem cells (MSC), vascular stromal fraction (VSF) and extracellular vesicles (EVs) as a promising treatment for OA [5]. These intra-articular injections have the advantages of high local bioavailability, fewer systemic side effects and lower cost compared to the surgery [6]. The majority of meta-analysis studies reported the superiority of PRP over HA [[7], [8], [9]]. However, Belk and co-workers have reported a short-term benefit after either HA or PRP treatment in their recent meta-analysis study [10]. Another recent study has reported no superiority of PRP when assessing joint structural changes [11]. Further high-quality multi-centre studies are warranted to prove clinical efficacy of HA and/or PRP, and to determine their long-term outcomes. On the other hand, MSC and VSF are still in the early phases of clinical trials to determine the optimum dose and their long-term side effects, while studies on EVs are still limited to in-vivo models [[12], [13], [14], [15]]. It’s noteworthy here that several meta-analysis studies reported the superiority of using MSC or VSF over PRP [5,16,17]. However, till now, the only option available to treat OA is total knee replacement surgery. This places OA among the most expensive conditions to treat [18,19]. Additionally, patients with comorbid conditions such as cardiovascular diseases are less suitable candidates for such intervention, and even if they undergo surgery, they will be at high risk of postoperative complications such as myocardial infarction and thrombosis [20,21]. Thus, it is crucial to elucidate pathogenesis, genetics as well as biomarkers of OA.

The increasing prevalence of obesity and a sedentary lifestyle nowadays, which are prominent risk factors for OA, resulted in a substantial increase in the number of people living with hip or knee OA [2]. Similarly to OA prevalence, obesity is dramatically increasing along with its accompanying consequences which places it at the top of public healthcare priorities. Unfortunately, the prevalence of obesity grew dramatically between 1975 and 2016 and this trend is still going on [22]. It is estimated that by the year 2030, about one billion people globally will be living with obesity [23]. As a matter of fact, the main burden of obesity lies in its interconnection with a plethora of other disorders. Obesity has always been distinguished as one of the most prominent and preventable risk factors for OA [22,24].

In this review, we aim to shed light on the different aspects of the obesity-OA relationship beyond the traditional theory of mechanical loading and to provide up-to-date information on the recently reported adipokines associated with OA in an attempt to highlight potential future therapeutic targets for OA. Before this, we will overview the difference between mechanical and metabolic theory for the obesity-OA relationship.

## 2 Mechanical load theory versus metabolic theory for explaining the obesity-OA relationship

Several authors showed a relationship between obesity and the risk of knee OA: for every 5-unit increase in body mass index (BMI), the associated increased risk of knee OA was 35% [25]. A cohort study in 2016 also showed that subjects with grade II obesity (BMI >35 kg/m2) were 4.7 times more likely to develop knee OA compared to subjects with normal weight [26]. Moreover, several reports showed that weight loss improved pain and function scores and decreased low-grade inflammation in patients with knee OA [[27], [28], [29], [30]]. For example, a study of overweight and obese adults with knee OA estimated that every pound (0.45 kg) of weight loss resulted in a four-fold reduction in the load exerted on the knee per step during daily activities, which appears to be clinically meaningful [31]. Similar findings were also reported in other studies [32,33]. The overloading on the joints could stimulate some inflammatory pathways such as (interleukin-1β (IL-1β), tumor necrosis factor α (TNF-α) and nuclear factor-kappa B (NF-κB)) leading to irreversible matrix degradation and apoptosis. It is worth mentioning that physiological mechanical loading is pivotal for healthy cartilage by inducing anabolic factors [34,35].

**Fig. 1:**
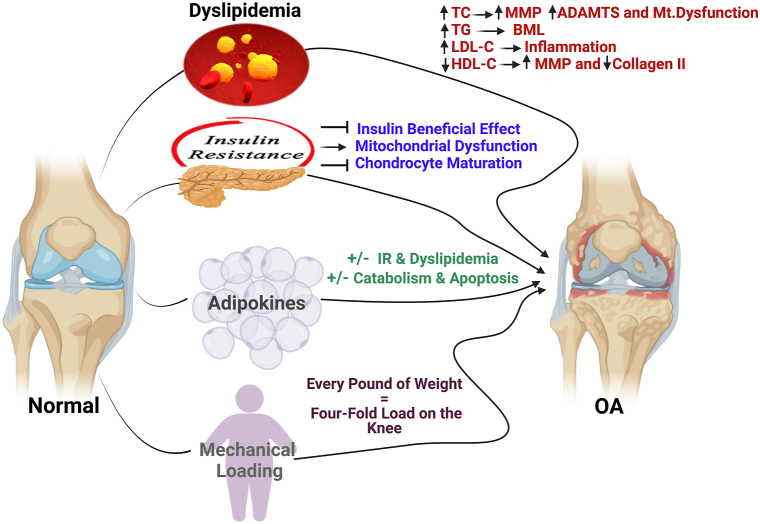
Multi-factorial interplay between obesity and OA. Dyslipidemia, insulin resistance, adipokines and mechanical loading are the four aspects by which obesity affects normal knee and induces OA. The arrows represent a summary of how each aspect leads to OA development. ADAMTS: a disintegrin and metalloproteinase with thrombospondin motifs, BML: bone marrow lesions, HDL-C: high-density lipoprotein cholesterol, IR: insulin resistance, LDL-C: low-density lipoprotein cholesterol, MMP: matrix metalloproteinases, Mt. dysfunction: mitochondrial dysfunction, OA: osteoarthritis, TC: total cholesterol and TAG: triglycerides. Created by Biorender.com.

Surprisingly, obesity has also been found to be strongly associated with hand and wrist OA, despite the fact that they are non-weight-bearing joints. That is why the mechanical loading hypothesis fails to explain the relationship between obesity and OA in these non-weight-bearing joints, which led to the postulation that adipose tissues play a paramount role in this context [36,37]. In fact, adipose tissues are a major source of cytokines, chemokines, and metabolically-active mediators known as “adipokines”. Some of these mediators are responsible for systemic low-grade inflammation associated with obesity, which establishes an environment promoting catabolic factors that degrade the joint [37]. However, others were reported to preserve the cartilage integrity and minimize osteophytes development [38,39]. Furthermore, obesity-related dyslipidemia and insulin resistance (IR) contributes significantly to the degenerative process [[40], [41], [42], [43]]. We tried to simplify the complex multi-factorial interplay between obesity and OA in Fig. 1.

## 3 Obesity-OA interplay beyond mechanical loading

### 3.1 Dyslipidemia and osteoarthritis

The relationship between dyslipidemia and OA is a controversial issue [44,45]. However, various studies revealed a significant relationship between lipotoxicity resulting from dyslipidemia and OA [41,43,46]. During OA, chondrocytes accumulate lipids, and their intracellular levels were reported to be correlated with the disease severity. Some scientists referred this to the change in the expression of genes involved in lipid efflux [47,48], while others highlighted some epigenetic modifications of cholesterol-related genes which are responsible for OA development [49]. Besides, several micro-RNAs were found to be associated with cholesterol dysregulation and hence OA [[50], [51], [52]]. Moreover, several animal studies unveiled a strong correlation between high-fat diet and the progression of OA via inducing synovial inflammation, formation of multiple osteophytes, systemic inflammation and metabolic changes [[53], [54], [55], [56]].

**Fig. 2:**
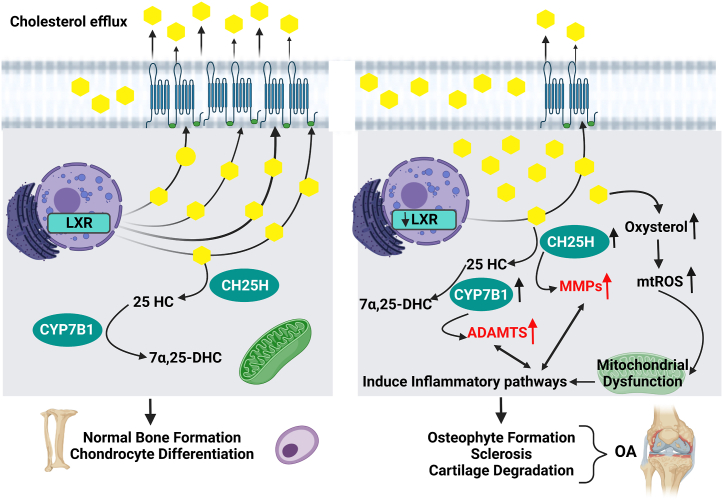
Hypercholesterolemia and OA interplay. During chondrocytes differentiation and bone formation, there is an increase in the expression of cholesterol efflux receptors. On the other hand, during OA, these receptors are downregulated that leads to cholesterol accumulation. Cholesterol hydroxylases are upregulated as well. As a result, degradative enzymes, catabolic factors and cholesterol byproducts increase leading to OA. ADAMTS: a disintegrin and metalloproteinase with thrombospondin motifs, CH25H: cholesterol 25-hydroxylase, CYP7B1: 25-hydroxycholesterol 7α-hydroxylase, 7α,25-DHC: 7α,25-dihydroxy cholesterol, 25-HC: 25-hydroxy cholesterol, LXR: liver X receptor, MMP: matrix metalloproteinases, mtROS: mitochondrial-reactive oxygen species, and OA: osteoarthritis. Created by Biorender.com.

In fact, cholesterol is a vital constituent of the cell membrane and controls signalling pathways in conjunction with other intermediates. In this context, cholesterol is essential for bone formation and chondrocyte differentiation. Cholesterol diffusion is regulated through cholesterol-influx genes: apolipoprotein-B (Apo-B) and cholesterol-efflux genes: Apo-A1, adenosine triphosphate binding cassette transporter-1 (ABCA-1) and liver X receptor (LXR), namely LXRα and LXRβ [49,57]. The expression of these receptors which are responsible for cholesterol efflux was reported to be increased during chondrocytes differentiation and decreased during hypertrophic state and hence during OA [47,58], as represented in Fig. 2. Interestingly, an in-vivo study revealed stimulation in inflammatory responses upon increasing intracellular cholesterol [59], whereas LXR agonists were found to prevent cholesterol accumulation and subsequent cartilage degeneration [49]. Besides the increase in cholesterol uptake in osteoarthritic cartilage, Choi and co-workers discovered an upregulation of cholesterol hydroxylases (mainly cholesterol 25-hydroxylase (CH25H) and 25-hydroxycholesterol 7α-hydroxylase (CYP7B1)). The upregulation of these enzymes leads to a decrease in the anabolic factors and induces synovitis, osteophyte formation and sclerosis. Additionally, in the same study, the high expression levels of CH25H were followed by high expression levels of various matrix metalloproteinases (MMP), while the increase in the expression of CYP7B1 resulted in an upregulation of ADAMTS (a disintegrin and metalloproteinase with thrombospondin motifs) [60], as shown in Fig. 2.

Other studies referred this causal relationship between cholesterol and OA to mitochondrial dysfunction. The increase in cholesterol oxidation products such as oxysterols were found to be associated with mitochondrial-oxidative stress resulting in an increase in mitochondrial-reactive oxygen species (mtROS). Overproduction of these mtROS activates oxidative stress and inflammatory pathways, which contribute to permanent damage of joint tissue [57], as shown in Fig. 2. Other different studies referred cholesterol-OA relationship to osteophyte formation rather than aggravation of cartilage damage [61]. It is noteworthy that several studies reported a protective effect of statins in both in-vitro and in-vivo OA models while others observed a low incidence of developing OA in patients receiving statins for a long time [[62], [63], [64], [65], [66]].

**Fig. 3:**
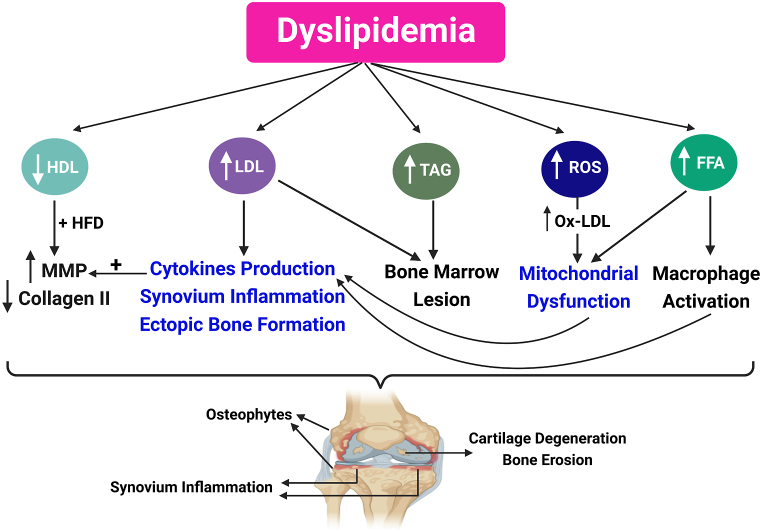
Multiple aspects of the contribution of dyslipidemia in the OA process. Dyslipidemia is characterized by high levels of LDL, TAG, FFA and ROS, and low levels of HDL. All these factors contribute to OA development and progression via several mechanisms. FFA: free fatty acids, HDL: high-density lipoprotein, HFD: high-fat diet, LDL: low-density lipoprotein, MMP: matrix metalloproteinases, Ox-LDL: oxidized low-density lipoprotein, ROS: reactive oxygen species, and TAG: triglycerides. Created by Biorender.com.

Moreover, in-vivo studies demonstrated a direct link between increased dietary cholesterol and OA development in Apo-A1 and LCAT (Lecithin–cholesterol acyltransferase) knockout mice [67]. In such models, the decrease in mature high-density lipoprotein (HDL) together with a high-fat diet was associated with an increase in MMP production and a decrease in collagen II and hence OA development [67], as shown in Fig. 3. It’s noteworthy here that this study was employed using LCAT(−/−) mice (having immature HDL and low HDL-cholesterol) and C57BL/6 mice (control mice having normal mature HDL). The authors in that study referred the lack of evidence of OA in the control group to the type and the duration of the high-fat diet administered. In contrast to other animal studies that unveiled a strong correlation between a high-fat diet and the progression of OA [53,54,68]; the energy from fats was very low (42% in Triantaphyllidou et al. study compared to 60% in other animal studies) and mice were placed on high-fat diet for a relatively shorter time (24 weeks in Triantaphyllidou et al. study, compared to 42 weeks in other animals studies) [67]. In addition, other in-vivo studies revealed an increase in cytokines production, synovium inflammation as well as ectopic bone formation arising from high low-density lipoprotein (LDL) levels [61,69]. It was also reported that hypercholesterolemia, hypertriglyceridemia and low HDL levels were associated with the occurrence of bone marrow lesions which is the source of pain during OA [46,48], represented in Fig. 3.

In fact, dyslipidemia is also characterized by an increase in free fatty acids (FFA) and reactive oxygen species (ROS) [70]. These ROS are responsible for the overproduction of oxidized-LDL (Ox-LDL) observed during dyslipidemia [71,72]. Both FFA and Ox-LDL participate in cartilage degeneration [[73], [74], [75], [76], [77]]. After binding to their receptors on chondrocytes, Ox-LDL either stimulate vascular endothelial growth factor (VEGF) release or affect the mitochondria. Consequently, the secretion of proteolytic enzymes and inflammatory cytokines increases leading to cartilage damage [75,78]. On the other hand, FFA activate macrophages and in turn macrophages start to secrete pro-inflammatory cytokines [48,57,79]. Besides, FFA induce mitochondrial dysfunction and IR which also participate in the degeneration process [42]. Fig. 3 summarizes these multiple aspects of the contribution of dyslipidaemia in the degenerative process in OA.

Recently, exploring and developing novel therapeutic avenues for the treatment of this lipotoxicity‐related OA has attracted many scientists [80,81]. A prime example of that is Sparstolonin B which has been identified as a novel therapy for lipotoxicity-induced OA. It prevents FFA from binding with their receptors; subsequently, it inhibits the NF-κB pathway and hence prevents cartilage degradation [81]. Another in-vitro study has reported that Firsocostat (ND-630) can attenuate lipid accumulation in chondrocytes by inhibiting acetyl co-A carboxylase (ACC; the key enzyme of de novo lipogenesis). Cartilage lipotoxicity was found to be associated with an up-regulation of ACC. Although Firsocostat is an investigational drug still under development, this study highlighted ACC inhibitors as a novel target for lipotoxicity-related OA [80].

### 3.2 Insulin resistance and osteoarthritis

**Fig. 4:**
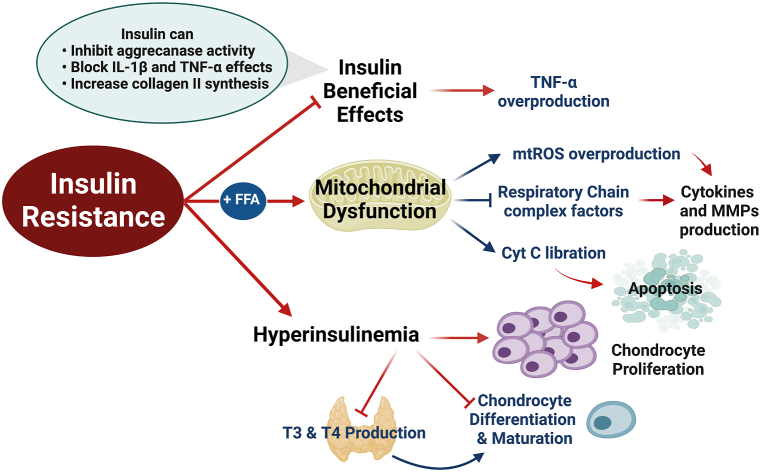
The role of insulin resistance in the OA degenerative process. During IR, the insulin receptors in chondrocytes and synovium become less responsive to insulin’s beneficial effects leading to the overproduction of cytokines. There is also an increase in FFA which leads to mitochondrial dysfunction which ultimately leads to cytokines release and apoptosis. Furthermore, hyperinsulinemia hinders the formation of mature chondrocytes by increasing the proliferation of chondrocytes, preventing their differentiation, and reducing the circulating levels of thyroid hormones. Red arrows with pointed ends imply the processes which are ultimately induced because of IR, while those with blunt ends imply processes which are ultimately inhibited as a consequence of IR. Cyt C: cytochrome C, FFA: free fatty acids, IL-1β: interleukin-1β, MMP: matrix metalloproteinases, mtROS: mitochondrial-reactive oxygen species, T3 and T4: thyroid hormones, and TNF-α: tumor necrosis factor α. Created by Biorender.com.

Several studies proved the role of insulin either as an anabolic or anti-catabolic factor. These studies reported that insulin can inhibit aggrecanase activity, block IL-1β and TNF-α effects as well as increase the synthesis of collagen II and other proteoglycans [[82], [83], [84], [85]]. Additionally, immunohistochemistry studies revealed that both synovium and chondrocytes possess insulin receptors [84,85]. Like other tissues, these receptors are also affected by the IR state in which they become less responsive to insulin’s beneficial effects [86]. In agreement with that, Hamada and co-workers reported that TNF-α expression is up-regulated in the synovium of diabetic OA patients when compared to non-diabetic OA patients [85]. They assigned these results to insulin-resistant state which rendered the synovium of diabetic patients more sensitive to overproducing local TNF-α in the absence of insulin regulatory effect [85], as shown in Fig. 4.

In fact, one of the hypotheses for the IR state is the increase of FFA [87]. Besides the aforementioned role of IR during OA, both FFA and IR can induce mitochondrial dysfunction leading to a reduction in the activity of complex factors of the respiratory chain, the liberation of cytochrome C as well as increased mtROS production [42,57,88]. As a consequence, the reduced activity of these complexes stimulates pro-inflammatory cytokine and MMP production inside chondrocytes, while cytochrome C initiates the apoptotic process [42,[88], [89], [90]], as discussed earlier. These concepts are presented in Fig. 4.

Moreover, another study investigated the effect of hyperinsulinemia, a characteristic feature in IR states, on the progression of OA. They reported that hyperinsulinemia increases the proliferation of chondrocytes and prevents their differentiation and maturation [91]. Furthermore, hyperinsulinemia reduces the circulating levels of thyroid hormones (T3 and T4) which are also necessary for chondrocytes maturation [91]. The role of hyperinsulinemia in the OA degenerative process is also presented in Fig. 4.

Another theory about how IR leads to OA is the hyperglycemic environment that results from IR. Glucose is necessary for articular chondrocyte to maintain cell homeostasis, produce energy and synthesize the cartilage matrix. However, hyperglycemia was found to increase inflammation, destruct subchondral bone and lead to chondrocyte dysfunction [92]. Previous studies revealed that ROS were found to be elevated in OA cartilage present in a hyperglycemic-like environment. As discussed before, ROS give rise to catabolic processes implicated in cell degradation and cell apoptosis [93]. Moreover, hyperglycemia is also known to favor the production of advanced glycation end products (AGE) and their accumulation in articular cartilage, which contribute to a toxic environment that might facilitate OA pathogenesis [93]. AGE then interact with the receptors of AGE (RAGE) and toll-like receptors (TLR), giving rise to a cascade of events that decrease the activation of peroxisome proliferator-activated receptor gamma (PPAR-γ), promote the release of pro-inflammatory factors such as TNF-α, and activate transcription factors such as NF-κB, which ultimately leads to inflammation, oxidative stress, and promotes cartilage degradation [93].

### 3.3 Adipokines and osteoarthritis

During the past years, adipokines have ignited much interest due to having either pro- or anti-inflammatory functions in various disorders [94]. Nowadays, adipokines are also believed to have significant implications in the pathogenesis and progression of obesity-induced OA [38,[95], [96], [97], [98]]. They can directly induce or inhibit catabolic or apoptotic pathways or affect bone metabolism [38,95,99]. For example, leptin, chemerin, resistin, visfatin and lipocalin-2 are now believed to have detrimental effects on joint tissue. However, others were reported to maintain cartilage integrity and reduce osteophyte formation, such as progranulin, vaspin and omentin-1 [38,39,95,[100], [101], [102], [103], [104], [105]]. Accordingly, adipokines have emerged as potential candidates to link obesity with OA which may also serve as putative targets for disease-modifying OA drugs, especially for obese patients [95,96,99,103,106]. Table 1 summarizes the reported adipokines interrelated with OA.

**Table 1:** The role of different adipokines in the pathogenesis of OA.

Adipokine	Role	References
**Leptin**	• Induces chondrocyte apoptosis by activating JAK2/STAT3 and mTOR pathways which stimulate ROS production. • Induces the secretion of MMP-1, MMP-3, MMP-13, ADAMTS-4, ADAMTS-5 and ADAMTS-9. • Activates JNK, MAPK, and NF-κB which Influence the synthesis of NO that induces inflammation via the expression of IL-6. IL-8 and PGE2. • Activates apoptosis process and inhibits autophagy via upregulation of LOXL3.	Zhang et al. [107], Zhao et al. [108], Jiang et al. [109] Hui et al. [110], Yaykasli et al. [111] Vuolteenaho et al. [112] Wei et al. [38]

**Chemerin**	• Recruits macrophages in synovium as part of the inflammatory cascade. • Induces CCL2, MMP-1, MMP-3 and MMP-13 expression. • Decreases the proliferative capability of chondrocytes. • Activates TLR4 and Akt/ERK pathways.	Eisinger et al. [113] Ma et al. [114] Wang et al. [104]

**Resistin**	• Binds to TLR4 and CAP1 receptors, which activate p38-MAPK and NF-κB, and C/EBP-β signalling pathways. • Promotes the release of pro-inflammatory cytokines/chemokines and degradative markers such as IL-6, IL-1α, IL-1β, TNF-α, CCL2, CCL3, CCL3L1, CCL4, CCL5, CCL8, CCL20, CXCL1, CXCL2, CXCL3, CXCL5, CXCL6, CXCL8 (IL-8), CX3CL1, MMP-1, MMP-13, and ADAMTS-4.	Zhang et al. [115] Lee et al. [116] Zhao et al. [105]

**Visfatin**	• Induces the production of IL-1β, IL-6, TNF-α, MMP-3, MMP-12, MMP-13, ADAMTS-4, and ADAMTS-5. • Induces osteophyte formation by inhibiting osteoclastogenesis.	Gosset et al. [117] Laiguillon et al. [118] Yang et al. [101] Beak et al. [119] Han et al. [120]

**Lipocalin-2**	• Reduces chondrocyte proliferation. • Forms a covalent complex with MMP-9 which blocks its auto-degradation.	Gupta et al. [39] Carrión et al. [95]

**Nesfatin**	• Induces pro-inflammatory mediators such as COX-2, IL-8, IL-6, and CCL3. • Stimulates IL-1β production via activating PI3K, Akt, AP-1 and NF-κB pathways which suppress miR-204–5p synthesis. • Conversely, another study identified nesfatin as an anti-inflammatory mediator which attenuates NF-κB and MAPK inflammatory pathways and induces collagen II expression.	Scotece et al. [121] Lee et al. [97] Jiang et al. [99]

**Adiponectin**	• Up-regulates TIMP-2 and inhibits IL-1β-mediated MMP13 production. • Induces autophagy by AMPK/mTOR activation.	Chen et al. [122], Xie et al. [123] Hu et al. [124], Duan et al. [106]

**Progranulin**	• Maintains the cartilage integrity by inhibiting ADAMTS-7 and ADAMTS-12 and by blocking the effect of TNF-α. • Inhibits IL-1β and LPS-induced catabolic pathways in chondrocytes by blocking TNFR1. • Triggers the anabolic pathways by binding to TNFR2. • Inhibits Wnt/catenin signalling with reduction of osteophyte formation and cartilage degeneration.	Guo et al. [125] Abella et al. [102] Zhao et al. [126] Zhao et al. [126], Carrión et al. [95]

**Vaspin**	• Reduces RANKL-induced expression of MMP-9. • Inhibits IL-1β and leptin-induced production of catabolic and pro-inflammatory mediators. • Its low level inhibits the expression of the cholesterol efflux pathway via miR155/LXRα led to cholesterol accumulation in cartilage. • Stimulates extracellular matrix anabolism and chondrocytes survival via activating Akt pathway.	Carrión et al. [95] Bao et al. [100,127] He et al. [52] Wang et al. [128]

**Omentin-1**	• Inhibits IL-1β-induced cartilage degradation via inhibition of the JAK2/STAT3 pathway which ameliorates MMP-1, MMP-3 and MMP-13 production. • Stimulates the mitochondrial biogenesis through PGC1α-AMPK signalling pathway. • Inhibits IL-1β-induced cellular senescence.	Li et al. [129] Li et al. [130] Chai et al. [103]

**Metrnl**	• Its high serum level was associated with a low risk for OA. • Records high levels in SF which suggested a compensatory role for it in SF. • Negative association between its SF levels and MMP-13 was found. • Suppress the PI3K/Akt/NF-κB pathway which upregulates collagen II and inhibits MMP-13 and ADAMTS-5. • Reduces chondrocyte pyroptosis by blocking the NLRP-3/caspase-1/GSDMD cascade.	Sobieh et al. [131] Liu et al. [132]

**Retinol Binding Protein 4 (RBP4)**	• Positive association was recorded between its level and MMP-1 and MMP-3.	Scotece et al. [133]

JAK/STAT3: Janus kinase/signal transducer and activator of transcription protein-3, mTOR: the mammalian target of rapamycin, ROS: reactive oxygen species MMP: matrix metalloproteinase, ADAMTS: a disintegrin and metalloproteinase with thrombospondin motifs, JNK: c-Jun N-terminal kinases, MAPK: mitogen activated protein kinase, NF-κB: nuclear factor-kappa B, NO: nitric oxide, IL: interleukin, PGE2: prostaglandin E2, LOXL3: Lysyl oxidase-like 3, CCL: C–C motif chemokine ligand, TLR4: toll-like receptor 4, Akt: protein kinase B, ERK: extracellular signal-regulated kinase, CAP1: adenylyl cyclase-associated protein 1, C/EBP-β: C/enhancer binding protein β, TNF-α: tumor necrosis factor α, CCL3L1: CCL3 like 1, CXCL: C-X-C motif chemokine ligand, CX3CL1: C-X3-C motif chemokine ligand 1, COX-2: cyclooxygenase-2, PI3K: Phosphoinositide-3-kinases, AP-1: activator protein 1, miR: micro-RNA, TIMP-2: tissue inhibitor matrix metalloproteinase 2, AMPK: adenosine monophosphate-activated protein kinase, LPS: lipopolysaccharide, TNFR: tumor necrosis factor receptor, RANKL: receptor activator of NF-κB ligand, LXRα: liver X receptor α, PGC1α: peroxisome proliferator-activated receptor gamma coactivator 1α, SF: synovial fluid, NLRP-3: nod-like receptor protein-3 and GSDMD: gasdermin D.

Surprisingly, adipose tissue is not the only source of adipokines production. Chondrocytes, synoviocytes as well as osteoblasts are also capable of producing certain adipokines, especially in arthritic patients [134,135]. Moreover, the infrapatellar fat pad, a loading force reducer which lies between the synovium and the joint capsule, has sparked a lot of curiosity because it is considered an intra-articular adipose tissue [19].

Since multiple reviews previously focused on the well-known adipokines and their role in OA in full details [95,96,98], currently this report will shed the light on only metrnl and retinol-binding protein 4 (RBP4) being emerging adipokines associated with OA, which are not only expressed by adipose tissue but also were found to be expressed by chondrocytes [131,133]. Few studies reported their association with OA with limited information about their exact molecular pathways. This review highlights the possible mechanism of action of metrnl and RBP4 in OA which will open the door for future experimental studies.

## 4 Emerging adipokines associated with OA

### 4.1 Metrnl

Metrnl was identified first as a protein homologous to the neurotrophin meteorin due to their sequence similarity, hence called meteorin-like (metrnl) [136]. However, unlike meteorin which is mainly expressed in the brain, this protein showed a relatively wider distribution in the body with higher expression levels in subcutaneous white adipose tissues and barrier tissues, and lower expression levels in the brain [136,137]. Metrnl is also called subfatin, IL-39 and cometin depending on its expression site and its possible effect as a cytokine [[136], [137], [138]]. Over the past years, several favourable actions for metrnl have been suggested. For instance, it can antagonize obesity-induced IR and improve glucose tolerance. This beneficial action is accomplished by the activation of PPAR-γ and by inducing protein kinase B (Akt) and adenosine monophosphate-activated protein kinase (AMPK) phosphorylation [[139], [140], [141]]. It is noteworthy to mention that PPAR-γ plays a critical role in the pathophysiology of OA; Its downregulation causes cartilage degeneration, chondrocyte apoptosis, and an upsurge of inflammatory mediators [142].

**Fig. 5:**
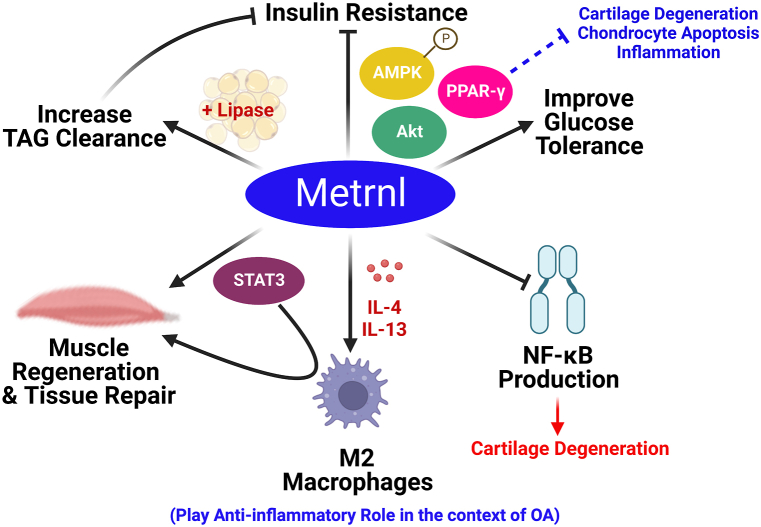
Metrnl beneficial effects as well as the reported/suggested pathways by which metrnl contributes to OA. Metrnl has several beneficial effects such as improving glucose tolerance and hence alleviating insulin resistance. This is mediated via the activation of PPAR-γ, Akt and AMPK. Metrnl has anti-inflammatory effects via inhibiting NF-κB signalling and activating M2 macrophages, the anti-inflammatory type. Additionally, Metrnl can regulate muscle regeneration and tissue repairs via the activation of STAT3 and M2 macrophages. Akt: protein kinase B, AMPK: adenosine monophosphate-activated protein kinase, IL: interleukin, NF-κB: nuclear factor-kappa B, PPAR-γ: peroxisome proliferator-activated receptor gamma, STAT3: signal transducer and activator of transcription protein-3, and TAG: triglycerides. Created by Biorender.com.

Additionally, metrnl enhances serum triglyceride clearance and elevates the expression and activity of lipase in adipose tissue. Increasing triglycerides turnover may be a possible mechanism by which metrnl alleviates IR [139]. Furthermore, increasing circulating metrnl in mice produced remarkable upregulation in the expression of genes associated with anti-inflammatory cytokines and can attenuate NF-κB mediated signalling [141,143]. Several immune-inflammatory disorders were associated with up-regulation in metrnl expression. Thus, metrnl was initially called IL-39 and changed later to IL-41 [137,144]. Metrnl can induce the expression levels of IL-4 and IL-13 which in turn stimulate M2 macrophages (alternative activated macrophages; the anti-inflammatory phenotype) [140]. Recently, several studies have reported the involvement of metrnl in balancing the inflammatory responses which encourages tissue repair and remodelling [[145], [146], [147]]. Baht and co-corkers referred metrnl’s ability to induce skeletal muscle regeneration to its ability to activate STAT3 (signal transducer and activator of transcription protein-3) signalling in muscle which in turn activates M2 macrophage polarization [145]. Other studies also support the pivotal role of STAT3 as an inflammatory regulator in different tissues [148,149]. However, in chondrocytes, STAT3 activation was found to induce degradation and apoptosis [150,151]. Thus, the role of STAT3 in OA pathogenesis, and its interrelation with metrnl seems to be a bit complicated and far from complete elucidation. Nevertheless, the context-specific relationship between STAT3 and the inflammatory pathways in different tissues is strongly suggested [149]. Therefore, future mechanistic molecular studies are indeed warranted to further elucidate the interplay between metrnl and STAT3 in OA models. All the previously discussed effects of metrnl are represented in Fig. 5.

Interestingly, significant up-regulation of metrnl expression was observed in synovial membranes of patients with rheumatoid arthritis (RA) [137]. Furthermore, metrnl expression was found to be elevated in hypertrophic chondrocytes [152]. Taking into consideration that hypertrophic chondrocytes happen to be a characteristic feature during OA pathogenesis [153] and that all the aforementioned pathways for metrnl were also found to be interconnected with OA pathogenesis, this strongly suggests a possible role of metrnl in OA pathogenesis.

Recently, we observed lower serum metrnl levels in obese patients with OA compared to obese patients not suffering OA, and higher serum metrnl levels were shown to be substantially linked with a reduced risk of OA. Furthermore, serum metrnl levels were shown to be lower in participants with advanced grade OA compared to subjects with early grade OA [131]. Regarding its synovial fluid (SF) levels, in that same study, it showed higher levels in obese patients with OA compared to obese subjects without OA. This led to the suggestion that there may be some sort of interplay between SF-metrnl and compensatory mechanisms in OA based on the previously reported favourable actions for metrnl (anti-inflammatory, insulin-sensitizing and its tissue repair role) [141,145,146,154]. This postulation was also based on the observed negative association between SF-metrnl and MMP-13 which is a well-known degradative marker during OA pathogenesis [131,155]. In agreement with our results, a recently published article has reported an association between the increasing level of metrnl and the alleviation of OA severity [132]. Liu and co-workers referred metrnl’s anti-inflammatory effect to the suppression of the PI3K (Phosphoinositide-3-kinases)/Akt/NF-κB pathway. This was associated by increasing in the expression of collagen II and inhibiting the expression of MMP-13 and ADAMTS-5. In the same study, metrnl reduced chondrocyte pyroptosis (the inflammatory form of programmed cell death) by blocking the nod-like receptor protein-3/caspase-1/gasdermin D cascade [132]. Collectively, these findings portray metrnl as a potential novel therapeutic target for OA and open the door for future research to further elucidate its mechanism of action in obesity-OA interplay [131].

### 4.2 Retinol binding protein 4 (RBP4)

RBP4 is well-recognized as an adipose tissue-derived hormone that belongs to the lipocalin family and promotes IR. It has also been identified as a vitamin A carrier in the blood responsible for carrying retinol, being a member of vitamin A family, from liver to peripheral tissues. Although RBP4 is mainly expressed by hepatocytes, it is also expressed by adipose tissues, and elevated RBP4 levels have been reported in obese humans as well as animal models [156,157]. Moreover, several studies documented a strong correlation between RBP4 and both IR and dyslipidemia; as it impairs glucose tolerance and insulin sensitivity and enhances the production of the gluconeogenic enzyme, phosphoenolpyruvate carboxykinase. In addition, low RBP4 levels or deletion of its gene have been linked to the enhancement of insulin sensitivity [156,[158], [159], [160], [161]]. It was reported that RBP4 impairs insulin signalling pathways either via the activation of PI3K/Akt or the activation of JAK2 (Janus kinase 2)/STAT5 [157,162]. It is noteworthy that the activation of PI3K/Akt or JAK signalling stimulates inflammation, catabolism and apoptosis in OA [97,109,129,132].

**Fig. 6:**
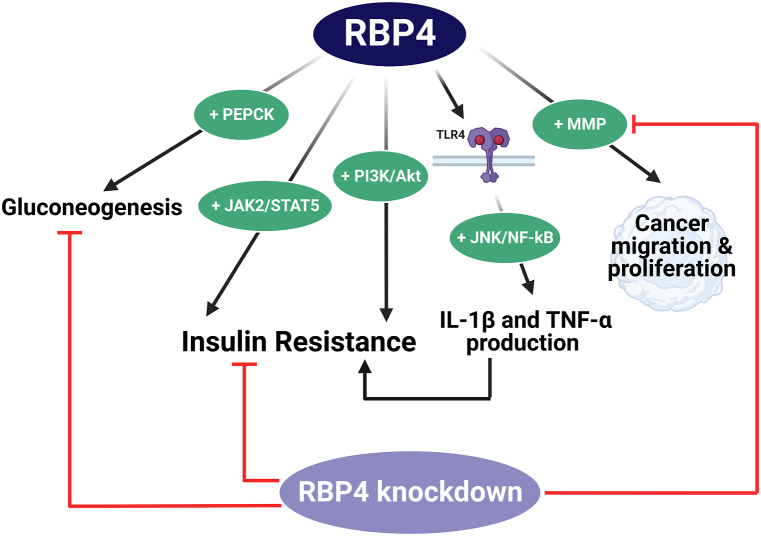
The reported effects of RBP4 by which it possibly contributes to OA pathogenesis. RBP4 induces IR by stimulating PI3K/Akt and JAK2/STAT5 pathways. In addition, RBP4 activates the inflammatory pathways (JNK and NF-κB) after binding with its receptor (TLR4). This leads to overproduction of inflammatory cytokines such as IL-1β and TNF-α. This also leads to IR development. RBP4 also induces gluconeogenesis by stimulating the gluconeogenic enzyme; PEPCK. RBP4 can promote MMP release in various tissues such as cancerous cells to enhance their migration and proliferation. Knockdown of RBP4 can suppress MMP production and gluconeogenesis and enhance insulin sensitivity. Akt: protein kinase B, IL-1β: interleukin-1β, IR: insulin resistance, JAK2/STAT5: Janus kinase 2/signal transducer and activator of transcription protein-5, JNK: c-Jun N-terminal kinases, MMP: matrix metalloproteinases, NF-κB: nuclear factor-kappa B, OA: osteoarthritis, PEPCK: phosphoenolpyruvate carboxykinase, PI3K: Phosphoinositide-3-kinases RBP4: retinol-binding protein 4, TLR4: toll-like receptor 4, and TNF-α: tumor necrosis factor α. Created by Biorender.com.

Importantly, RBP4 binds to either retinoic acid gene homologous 6 receptor or TLR4 [157]. After binding to TLR4, RBP4 stimulate c-Jun N-terminal kinases (JNK) and NF-κB, which subsequently stimulate IL-1β and TNF-α production leading to insulin signalling impairment and hence IR state develops [157]. Interestingly, TLR activation leads to the production of pro-inflammatory cytokines in chondrocytes as well [163]. This partially explains the suggested contribution of RBP4 in OA pathogenesis, together with the latest investigations which have revealed that RBP4 can promote MMP release to promote cancerous cells migration and proliferation and that the knock-down of RBP4 suppresses the production of MMP [133,164,165]. The previously suggested mechanisms for the involvement of RBP4 in OA are presented in Fig. 6.

Recently, Scotece and co-workers have investigated the potential role of RBP4 in OA pathogenesis, and they found that RBP4 is expressed by osteoarthritic chondrocytes and at the same time its receptor was also detected [133]. In addition, a positive correlation was observed between RBP4 and the classic degradative markers MMP-1 and MMP-3. This study has exhibited RBP4 as a novel target in OA that connects IR, inflammation and cartilage degeneration [133].

## 5 The inter-relation between various metabolic aspects in OA reveals possible therapeutic targets

Dyslipidemia, insulin resistance and adipokines could affect the cartilage tissue by acting alone or in a synergistic way. Adipokines act as key players in the interplay between obesity and OA. Dysregulation of adipokines levels is responsible for developing IR and dyslipidemia [96], as shown in Fig. 1. Another possible key factor is the mitochondrial dysfunction resulting from hypercholesterolemia and IR. Mitochondrial dysfunction leads to stimulating inflammatory and catabolic pathways, as well as chondrocyte apoptosis [42,57,88,89], as shown in Figs. 2 and 4. In agreement with that, Coleman and co-workers support targeting mitochondrial responses in ameliorating OA. They proved that Amobarbital or N-acetylcysteine can inhibit oxidative stress after injury and protects from posttraumatic OA development [166]. Additionally, coenzyme Q10 (CoQ10) has been suggested to slow OA progression and enhance OA symptoms. CoQ10 is an anti-oxidant and an important factor in the mitochondrial respiratory chain. It could also modulate the inflammatory pathways such as NF-κB and TLR, and control the release of pro-inflammatory cytokines in different disorders [167,168]. In this context, a recent in-vivo study has reported the effect of CoQ10-micelles as oral supplements in ameliorating the inflammation associated with OA [169]. Similar findings were also reported about melatonin, dihydromyricetin, quercetin, taurine, resveratrol and diallyl disulfide. These anti-oxidants could be potential treatment for OA by protecting chondrocytes from mitochondrial oxidative stress [[170], [171], [172], [173], [174], [175], [176]]. However, clinical trials are still needed to prove such effects in OA patients.

Another interrelation between dyslipidemia, insulin resistance and adipokines is their abilities to induce inflammatory and catabolic pathways [42,60,85,94]. Thus, anti-cytokines and enzyme inhibitors are also suggested as potential therapy for OA such as TNF-inhibitors, IL-6 antibodies, IL-1β antibodies, MMP inhibitor and JAK inhibitors [177]. Some investigational studies on RA reported the role of anti-TNF therapy in enhancing IR and modulating adipokines levels such as resistin, chemerin and adiponectin [[178], [179], [180]]. However, these kinds of therapies are still in their early phases with severe adverse effects [177].

Till the discovery of a disease-modifying OA drug, a focus on prevention is valuable. Individuals' ability to self-monitor their weight, diet and physical activity is a complicated process that is influenced by their level of education and access to information. Moreover, it consumes time and effort to achieve change. Solutions that are focused on improved nutrition and enhancement of physical activity could be only successful if individuals receive personalized information that can be transformed into practical activities with a result that can be measured and related to their physiological status [181,182]. Over the last decade, technological applications and devices have aided in the maintenance of healthy behaviors. Web-based weight loss intervention programs helped many people to reduce their BMI scores [[182], [183], [184]]. For example, ArmOnIA is an online digital tool that incorporates dietary, anthropometric, and physical activity data and produces an individual’s estimation of energy balance [182]. Another personalized model is called personalized metabolic avatar (PMA). This model can predict the individuals’ responses to diet, physical activity and environmental and psychological factors. Thus, PMA could evaluate each diet plan and help to reach the ideal BMI [184]. Both ArmOnIA and PMA can raise public self-awareness and help people pursue a healthy life and observe long-lasting outcomes [182,184].

## 6 Conclusion

OA is no longer considered to be a tear and wear form of arthritis. To reduce its burden and improve patients’ life, discovering new biomarkers and therapeutic targets is crucial to improving diagnosis and treatment. This necessitates a more thorough understanding of the various molecular mechanisms governing the OA disease process from the very beginning and through the development of joint degenerative deterioration. Obesity is interrelated with OA pathogenesis and contributes to the disease process via multiple connection points, namely, dyslipidemia, IR, and adipokines. Several adipokines have been reported to be associated with OA. Particularly, metrnl and RBP4, not only have been reported to be associated with OA, but also, they are produced by chondrocytes. This sheds light on their strong potential to provide novel therapeutic targets for OA, and also highlights that further studies are warranted to further elucidate their molecular mechanisms in OA. Conclusively, several emerging findings establish OA as a metabolic disease and highlight the metabolic contribution of obesity in OA pathogenesis rather than the traditional mechanical-loading theory. Careful consideration for the various molecular and metabolic mechanisms interrelated with obesity-OA interplay will undoubtedly unveil a plethora of novel therapeutic targets and new avenues for OA treatment.

## Author contribution statement

All authors listed have significantly contributed to the development and the writing of this article.

## Funding statement

This research did not receive any specific grant from funding agencies in the public, commercial, or not-for-profit sectors.

## Data availability statement

Authors confirm that all relevant data are included in the paper and/or supplementary information files.

## Declaration of interest’s statement.

The authors declare no competing interests.

## Abbreviations

OA: osteoarthritis
HA: hyaluronic acid
PRP: platelet-rich plasma
MSC: mesenchymal stem cells
VSF: vascular stromal fraction
EVs: extracellular vesicles
BMI: body mass index
IL: interleukin
TNF-α: tumor necrosis factor α
NF-κB: nuclear factor-kappa B
IR: insulin resistance
LXR: liver X receptor
CH25H: cholesterol 25-hydroxylase
CYP7B1: 25-hydroxycholesterol 7α-hydroxylase
MMP: matrix metalloproteinase
mtROS: mitochondrial-reactive oxygen species
LCAT: lecithin–cholesterol acyltransferase
HDL: high-density lipoprotein
LDL: low-density lipoprotein
FFA: free fatty acids
ROS: reactive oxygen species
Ox-LDL: oxidized-LDL
VEGF: vascular endothelial growth factor
ACC: acetyl co-A carboxylase
AGE: advanced glycation end product
RAGE: receptors of AGE
TLR: toll-like receptors
PPAR-γ: peroxisome proliferator-activated receptor gamma
RBP4: retinol-binding protein 4
metrnl: meteorin-like
Akt: protein kinase B
AMPK: adenosine monophosphate-activated protein kinase
STAT3: signal transducer and activator of transcription protein-3
RA: rheumatoid arthritis
SF: synovial fluid
PI3K: Phosphoinositide-3-kinases
ADAMTS: a disintegrin and metalloproteinase with thrombospondin motifs
JAK: Janus kinase
JNK: c-Jun N-terminal kinases
CoQ10: coenzyme Q10
PMA: personalized metabolic avatar
